# *Haemophilus influenzae* type a as a cause of paediatric septic arthritis

**DOI:** 10.1099/jmmcr.0.005064

**Published:** 2016-10-27

**Authors:** Joelle Thorgrimson, Marina Ulanova

**Affiliations:** Northern Ontario School of Medicine, 955 Oliver Road, Thunder Bay, ON P7B 5E1, Canada

**Keywords:** *Haemophilus influenzae*, septic arthritis, indigenous populations

## Abstract

**Introduction:**

Incidence rates of invasive *Haemophilus influenzae* serotype b disease have decreased significantly since the introduction of the Hib vaccine; however, the rates in indigenous populations remain disproportionately high, specifically in the paediatric population. Additionally, with the decline of type b invasive infections, there has been a rebound in the incidence of invasive infections caused by other strains of *H. influenzae*, particularly serotype a.

**Case presentation:**

We present a paediatric case of septic arthritis caused by *H. influenzae* type a in a toddler that was fully resolved following antibiotic therapy. This report adds to other reports of septic arthritis in indigenous populations as shown through a review of recently documented *H. influenzae* type a septic arthritis cases.

**Conclusion:**

Socio-economic risk factors for invasive *H. influenzae* type a disease, such as poverty, poor housing conditions, overcrowding, smoking and substance abuse during pregnancy, as well as the need for *H. influenzae* type a immunization of vulnerable populations, are discussed.

## Introduction

*Haemophilus influenzae* is a Gram-negative coccobacillus that exists as encapsulated and unencapsulated (non-typeable) strains. The encapsulated strains carry a unique polysaccharide capsule, and they are correspondingly divided into six serotypes, namely a–f (Pittman, 1931). Among *H. influenzae* serotypes, invasive *H. influenzae* type b disease has the most severe manifestations (Wenger, 1998). *H. influenzae* type b was a major cause of paediatric invasive bacterial disease in Canada until the early 1990s when the Hib vaccine was introduced, which resulted in a dramatic decrease in the incidence rates of *H. influenzae* type b infections (Wenger, 1998; Adam *et al.*, 2010). In the post-Hib vaccine era, increased incidence rates of invasive disease caused by non-b serotypes of *H. influenzae* have been documented in several countries, including Canada, that could possibly be due to the unmasking of less prevalent strains or the result of more systematic surveillance (Adam *et al.*, 2010; Bruce *et al.*, 2008; Ladhani *et al.*, 2010; Millar *et al.*, 2005) An increase in incidence rates of invasive *H. influenzae* type a disease has recently been documented in certain geographical areas characterized by a high proportion of indigenous people, such as the North American Arctic (reviewed by Tsang *et al.*, 2014). A recent emergence of invasive *H. influenzae* type a disease was reported in Alaska, where it had not been identified prior to 2002 despite the consistent surveillance of invasive *H. influenzae* disease (Bruce *et al.*, 2013).

The rebound of *H. influenzae* type a is of significant concern in indigenous populations, because the incidence of *H. influenzae* infections has historically been higher in indigenous children than in the general population; the rates in indigenous children remain higher even with the overall decrease in infections after the Hib vaccine was implemented (Ulanova & Tsang, 2014). Research is currently being carried out to determine why indigenous populations in North America are more prone to these infections and whether a *H. influenzae* type a vaccine may be required to control invasive *H. influenzae* type a disease in susceptible populations (Desai *et al.*, 2014).

Although *H. influenzae* is commonly carried in the nasopharynx asymptomatically, it is capable of causing local and invasive infections such as otitis media, sinusitis, pericarditis, urinary tract infections, septicaemia, septic arthritis, meningitis, pneumonia and epiglottitis (Pittman, 1931; Wenger, 1998). We describe a case of a paediatric patient who presented with fever, pain, erythema and swelling of the left foot. This patient was consequently diagnosed with septic arthritis caused by *H. influenzae* type a.

## Case report

A 3-year-old girl presented to a local emergency department with pain and tenderness of the dorsum of the left foot after being stepped on by a sibling 12 h prior. Her past medical history was significant for pre-term birth at 23 weeks, for which she spent 3 weeks in a tertiary care intensive care unit, and foetal alcohol syndrome. She also had intermittent asthma and a history of acute otitis media. Her immunizations were up to date and she was in the care of indigenous family services.

The patient was in the eighth percentile for weight. She was febrile at 38.8 °C and had borderline tachycardia of 140 beats min^−1^. Her oxygen saturation and respiration rate were within normal limits. On examination, the patient was reluctant to move her foot. Pedal pulses were palpable. No skin rashes were observed. No enlarged lymph nodes were found in femoral or neck regions. Tympanic membranes were intact with no signs of effusion. The throat was clear with no exudate, swelling or deviated uvula. There were no adventitious heart or lung sounds. The abdomen was soft and non-tender with bowel sounds present. The cranial nerve examination and peripheral nerve examination were normal.

Given the history of possible trauma, x-rays were performed of the left foot; no abnormalities or bony injuries were noted. Blood tests revealed a haemoglobin level of 125 g l^−1^ (normal=112–165 g l^−1^), a white blood cell count of 11.2×10^9^cells l^−1^ (normal=4.7–10.3×10^9 ^cells l^−1^) and an alkaline phosphatase level of 380 IU l^−1^ (normal=115–375 IU l^−1^). Blood cultures were collected. A concern for cellulitis and/or septic arthritis was raised. The patient was admitted to the hospital for treatment and observation on intravenous (IV) cefazolin (400 mg every 8 h), oral ibuprofen (200 mg every 4–6 h as needed) and oral codeine (15 mg every 4 h as needed).

The following day, blood culture results demonstrated the growth of *H. influenzae* type a sensitive to ampicillin, chloramphenicol and cefotaxime, with no detection of β-lactamase enzymes. The antibiotic was switched to IV cefotaxime (500 mg every 8 h). Cerebral spinal fluid (CSF) samples were collected; the CSF indices were normal with sterile cultures.

Limited improvement was seen the following day, so the patient was sent to a tertiary care facility for a bone scan. The scan showed increased flow in the left foot, specifically in the base of the left first metatarsal. On delayed images, focal increased activity was seen in the base of the first metatarsal and the first cuneiform. The findings were consistent with septic arthritis of the first tarsometatarsal joint with bone oedema on either side of the joint. Two days later, the cefotaxime dose was increased to 750 mg every 8 h (150 mg kg^−1 ^daily).

The patient remained in hospital for 9 days. During this time the erythema and swelling improved, followed by decreased pain. Upon discharge, the left dorsum of the foot was still tender but the patient was able to walk. The patient was discharged with IV cefotaxime (750 mg every 8 h) with plans to follow up with a paediatrician in 1 week and to continue antibiotics for 6 weeks in total. The patient made a full recovery and had not had any recurrences since this time.

## Discussion

This report demonstrates that in the era of universal paediatric immunization against *H. influenzae* type b, type a is able to cause severe paediatric invasive disease, which is reminiscent of type b in the pre-Hib vaccine time. Experimental animal studies demonstrated that among encapsulated *H. influenzae* strains, type b was the most virulent, followed by type a, and serotypes c–f were less virulent (Zwahlen *et al.*, 1989). The Hib vaccine success decreased the incidence of invasive *H. influenzae* type b infections, particularly in paediatric populations. However, during the last two decades, cases of invasive *H. influenzae* type a disease have been reported in infants and toddlers (reviewed by Ulanova & Tsang, 2014). Recently, the presence of anti-*H. influenzae* type a antibodies has been documented in cord blood sera (Schmidt *et al.*, 2012). Although newborns can be protected against invasive bacterial infections by transplacentally acquired antibodies, during the first 2 months of life the maternal antibodies decline limiting protection to the infant. In Canada, the Hib vaccine is first given at the age of 2 months, followed by doses at 4 and 6 months, and a booster at 12 months of age, which induces protective antibody levels in infants. As a result of the publicly funded immunization program, the proportion of *H. influenzae* infections caused by different strains has changed, with a decrease in invasive *H. influenzae* type b and an increase in other serotypes and non-typeable *H. influenzae* (Tsang *et al.*, 2014).

Specifically in indigenous populations, serotype a is now the most common cause of invasive *H. influenzae* disease, including paediatric septic arthritis (Bruce *et al.*, 2008, 2013; Tsang *et al.*, 2014). Paediatric cases of septic arthritis caused by *H. influenzae* type a have been reported from Canada, USA, Brazil and Australia (Table 1). Paediatric septic arthritis is a common presentation of invasive *H. influenzae* type a disease in indigenous people of the Canadian Arctic (Li *et al.*, 2016). According to recent data by Pavlik and co-authors, in New Mexico (2003–2014), *H. influenzae* type a was responsible for 8 out of 10 cases of paediatric septic arthritis caused by non-type b *H. influenzae*, and Native Americans were overrepresented among the cases (5; 50 %), although Native American children constituted 15.8 % of paediatric patients admitted to the hospital (Pavlik *et al.*, 2016). These cases of *H. influenzae *type a septic arthritis showed remarkable similarities with our case, i.e. the young age (10–36 months), and the dominant localization in the lower limbs (Pavlik *et al.*, 2016). To our knowledge, the case presented here is the second report of invasive *H. influenzae* type a disease causing septic arthritis in Northwestern Ontario, Canada.

**Table 1.** Previous published cases of paediatric septic arthritis caused by *H. influenzae* type a

Reference	Location	Age (months)	Ethnicity	Sex	Clinical presentation	Disease outcome	Underlying condition	Immunization	*H. influenzae* type a isolate site
Hammitt *et al.* (2005)	Western Alaska, USA	8	Alaskan native	Male	1 day history of fever and refusal to use left leg	Recovery with treatment of ceftriaxone; recurrent episode 3 months later in left leg and left arm, which recovered with ceftriaxone and vancomycin	History of pyelonephritis	Up to date	Joint fluid and blood
Kapogiannis *et al.* (2005)	Georgia, USA	30	African American	Male	5 day history of fever and 3 day history of decreased range of motion of right hip and neck	Treated with cefotaxime; recovered, but had significant cognitive and motor developmental deficits and bilateral sensorineural hearing loss	None	None	Joint fluid, blood, CSF
Bruce *et al.* (2008)	Alaska, USA	<12	Not reported	Not reported	Septic arthritis	Recurrent disease 4 months later	None	Not reported	Not reported
De Almeida *et al.* (2008)	Rio de Janeiro, Brazil	36	Not reported	Female	Fever, severe leg pain, restricted hip joint motility and motor difficulties	Treated with cefuroxime; full recovery	None	Up to date	Joint fluid
Kelly *et al.* (2011)	Northwestern Ontario, Canada	33	Indigenous	Male	Fever with swollen, erythematous, painful right ankle	Treated with cefuroxime and ampicillin; full recovery	None	Not reported	Blood
Fischer (2014)	Rural remote Australia	9	Indigenous	Male	2 day history of irritability and distress on movement of the left leg; afebrile	Treated with cefotaxime; full recovery	None	Up to date	Joint fluid, blood
Pavlik *et al.* (2016)	New Mexico, USA	13	Indigenous	Male	Ankle symptoms 1 day before presentation	Not reported	Not reported	Not reported	Joint fluid
		12	Latino	Male	Ankle symptoms 4 days before presentation; bone involvement				Joint fluid
		36	Indigenous	Female	Hip symptoms 5 days before presentation				Joint fluid
		36	Latino	Male	Ankle symptoms 7 days before presentation; bone involvement				Joint fluid, blood
		76	Latino	Male	Ankle symptoms 2 days before presentation; bone involvement				Joint fluid
		22	Indigenous	Male	Knee symptoms 5 days before presentation				Joint fluid
		10	Indigenous	Female	Hip symptoms 5 days before presentation				Joint fluid
		10	Indigenous	Female	Ankle symptoms 2 days before presentation				Joint fluid, blood

The most common mechanism of infection spread in septic arthritis is haematogenous. With no evidence of penetrating trauma prior to presentation, it may be assumed that carriage of *H. influenzae* type a preceded the disease, e.g. in the nasopharynx. This patient reported being stepped on by a sibling 12 h prior to the development of clinical symptoms. Although non-penetrating trauma has not been documented as a risk factor for septic arthritis (Frederiksen *et al.*, 1993; Kaandorp *et al.*, 1995), it is possible that this event was causally related. The trauma may have caused a non-specific pro-inflammatory response, which increased capillary permeability and facilitated *H. influenzae* type a dissemination within the joint. In this case, other relevant risk factors that may potentially have had a negative impact on immune responses included prematurity and foetal alcohol syndrome (Caird *et al.*, 2006; Gauthier, 2015).

An increased susceptibility of indigenous children to invasive *H. influenzae *type a disease may be due to the widely recognized unfavourable socio-economic factors present in indigenous communities. In the Arctic, an augmented transmission rate of *H. influenzae* was reported to be associated with smoking in pregnancy, prematurity, lack of breastfeeding, shared care with more than one child younger than 2 years of age, adoption status, Inuit ethnicity, as well as with wood heating, rodents in the home, livestock near the home, overcrowding and rural residence (Banerji *et al.*, 2009; Hennessy *et al.*, 2008). In Navajo children, poor housing conditions, such as a lack of an in-home water service, were reported to increase *H. influenzae* type b infection rates (Wolff *et al.*, 1999). Similar socio-economic factors underlying an increased susceptibility to invasive *H. influenzae* disease are commonly present in various indigenous populations (Tsang *et al.*, 2014). Indeed, Native American children were prevalent among paediatric cases with septic arthritis caused by all *H. influenzae* strains in New Mexico (Pavlik **et al.*,* 2016). Before an accurate comparison can be made between differing ethnicities, socio-economic factors must be taken into account.

A new *H. influenzae* type a vaccine is currently being developed in Canada (by the National Research Council, Public Health Agency of Canada and Northern Ontario School of Medicine) in collaboration with the U.S. Centers for Disease Control and Prevention, and it is anticipated that it may significantly alleviate the burden of this disease in vulnerable populations similar to what has happened as a result of universal paediatric immunization against *H. influenzae* type b (Desai *et al.*, 2014). However, before this vaccine can become available for immunization of vulnerable populations several important challenges have to be addressed. Although previous experience with the development of conjugate Hib vaccine is highly relevant considering the similarities in the immunological characteristics and natural history of invasive *H. influenzae* type a and type b diseases, more research is needed with regards to *H. influenzae* type a. In particular, immunological correlates of protection against *H. influenzae* type a infection have yet to be established. Given that indigenous children contract invasive *H. influenzae* type a disease at an early age, the choice of a carrier protein for the polysaccharide-protein conjugate is critically important to achieve timely formation of protective immunity as emphasized by Tsang *et al*. (2014). Indeed, the use of *Neisseria meningitidis* group B outer membrane protein (OMP) as a carrier for the *H. influenzae* type b capsular polysaccharide antigen was demonstrated to induce a protective anti-*H. influenzae* type b immunity in 2-month-old indigenous children that could not be achieved with the use of other carrier proteins (reviewed by Tsang *et al.*, 2014).
